# Supplementary material for: Nursing students’ perceived anxiety and heart rate variability in mock skill competency assessment
Source: PLoS One. 2023 Oct 26;18(10):e0293509. doi: 10.1371/journal.pone.0293509 (PMC10602303; doi:10.1371/journal.pone.0293509)
Supplement: S1 Fig — Each node shows the sample average rank of HRV. (DOCX) [file pone.0293509.s001.docx]

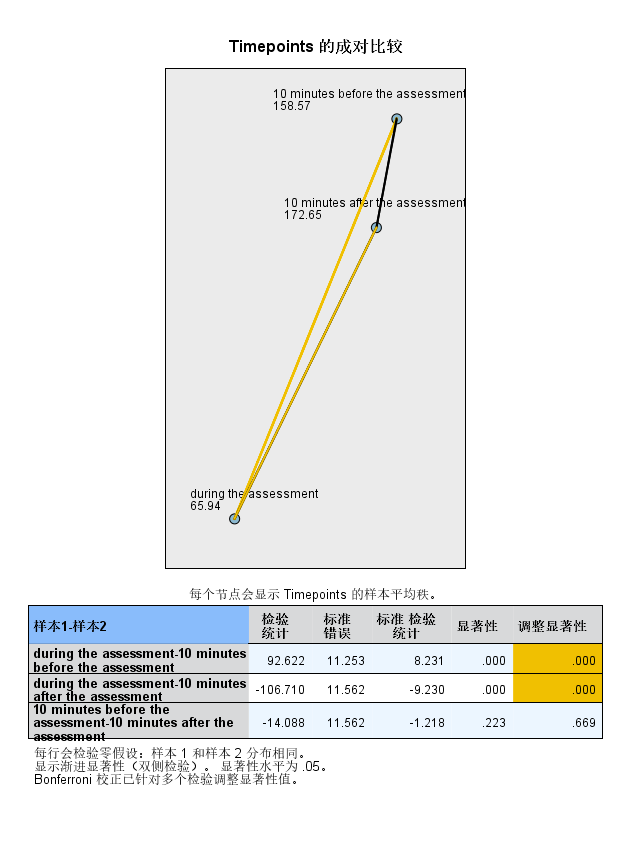


**S1 Fig A. Pairwise comparisons of three time points for all participants.**

Each node shows the sample average rank of HRV.


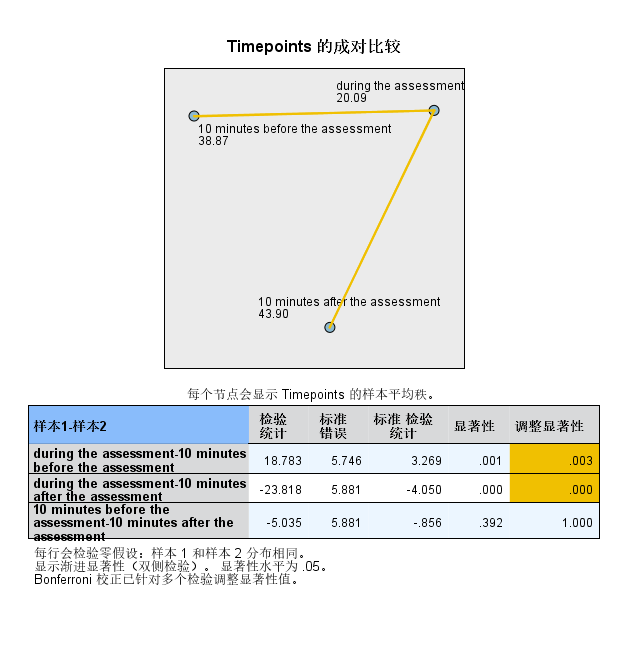


**S1 Fig B. Pairwise comparisons of three time pointss for male participants.**

Each node shows the sample average rank of HRV.


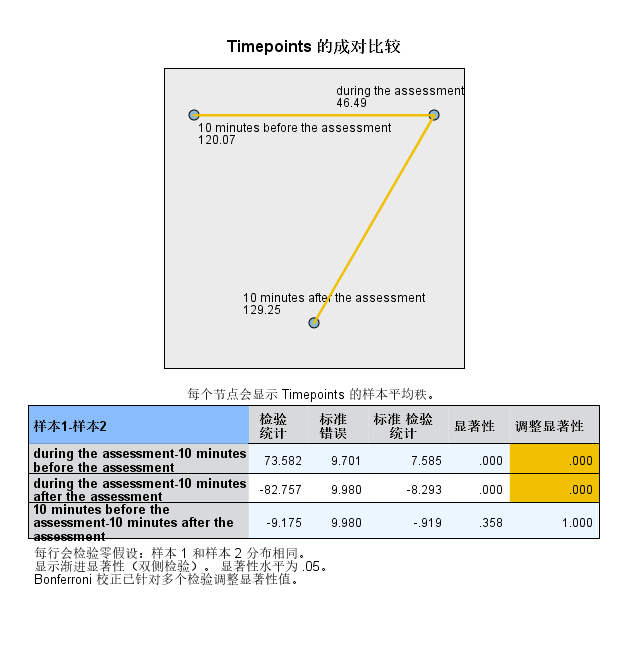


**S1 Fig C. Pairwise comparisons of three time points for female participants.**

Each node shows the sample average rank of HRV.
