# Supplementary material for: Nursing students’ perceived anxiety and heart rate variability in mock skill competency assessment
Source: PLoS One. 2023 Oct 26;18(10):e0293509. doi: 10.1371/journal.pone.0293509 (PMC10602303; doi:10.1371/journal.pone.0293509)
Supplement: S2 Fig — Each node shows the sample average rank of performance. (DOCX) [file pone.0293509.s002.docx]

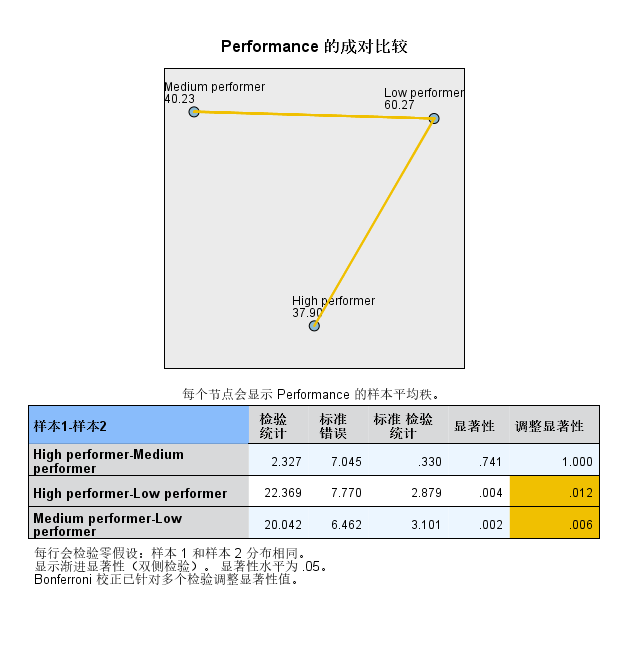


**S2 Fig A. Pairwise comparisons of performance level during the assessment (T1)**

Each node shows the sample average rank of performance.


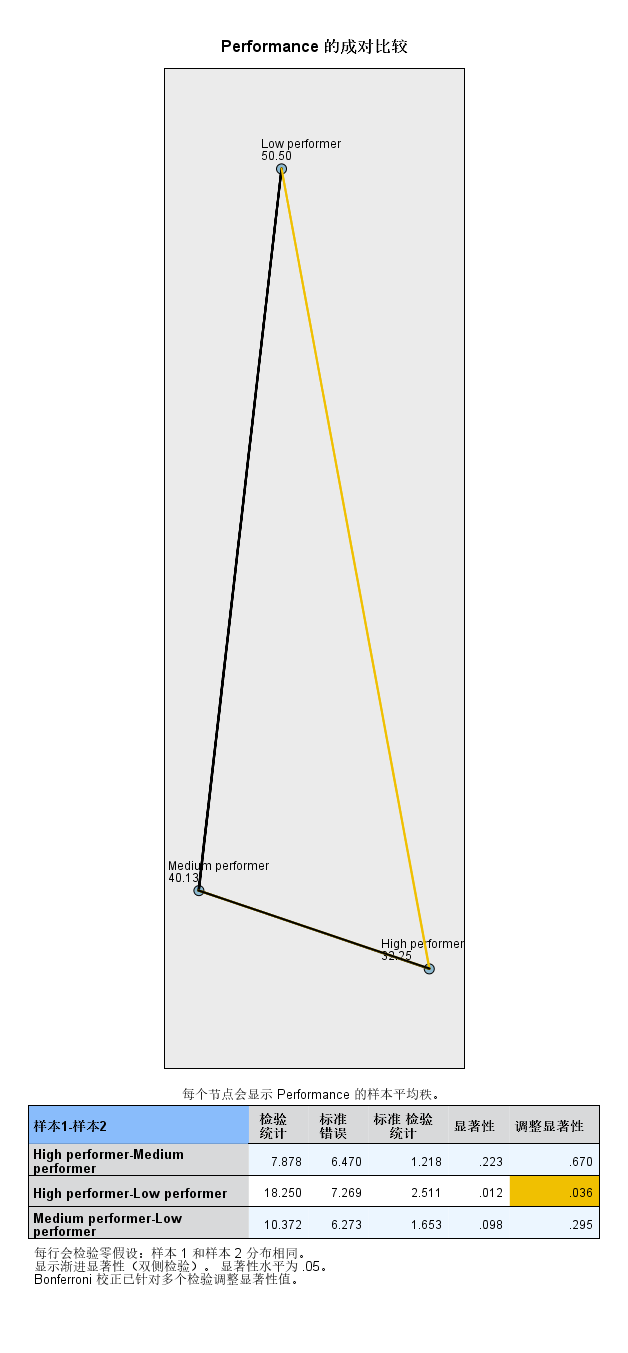


**S2 Fig B.** **Pairwise comparisons of performance level 10 minutes after the assessment (T2)**

Each node shows the sample average rank of performance.
