# Supplementary material for: Nursing students’ perceived anxiety and heart rate variability in mock skill competency assessment
Source: PLoS One. 2023 Oct 26;18(10):e0293509. doi: 10.1371/journal.pone.0293509 (PMC10602303; doi:10.1371/journal.pone.0293509)
Supplement: S3 Fig — Each node shows the sample average rank of performance. (DOCX) [file pone.0293509.s003.docx]

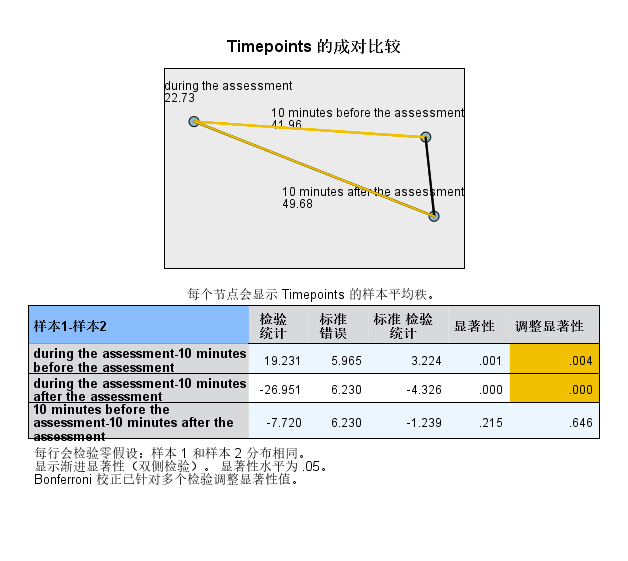


**S3 Fig A.** **Pairwise comparisons of three time points for low performers.**

Each node shows the sample average rank of performance.


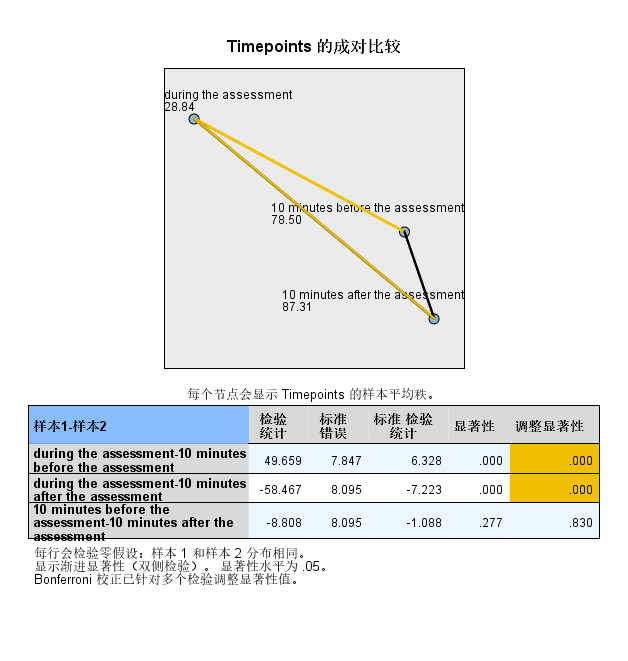


**S3 Fig B. Pairwise comparisons of three time points for medium performers.**

Each node shows the sample average rank of performance.


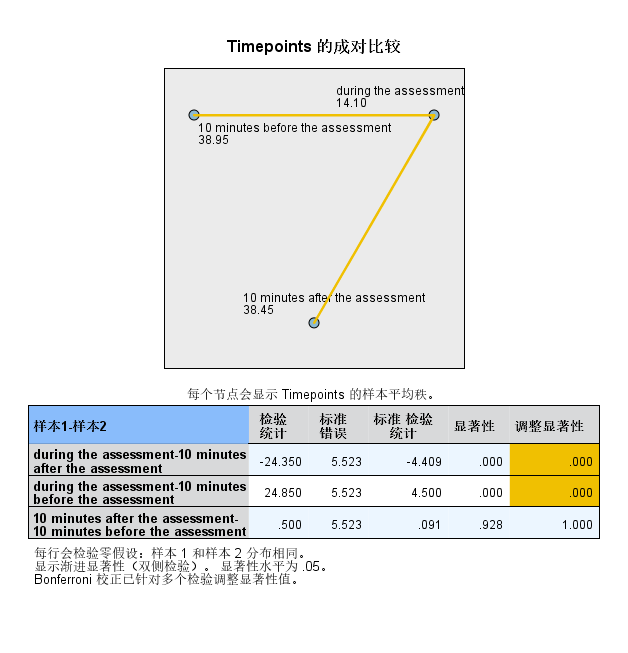


**S3 Fig C. Pairwise comparisons of three time points for high performers.**

Each node shows the sample average rank of performance.
