# Supplementary material for: Nursing students’ perceived anxiety and heart rate variability in mock skill competency assessment
Source: PLoS One. 2023 Oct 26;18(10):e0293509. doi: 10.1371/journal.pone.0293509 (PMC10602303; doi:10.1371/journal.pone.0293509)
Supplement: S1 Table — Each row tests the null hypothesis that the Sample 1 and Sample 2 distributions are the same. Asymptotic significances (2-sided tests) are displayed. The significance level is 0.05. (DOCX) [file pone.0293509.s004.docx]

**S1 Table.** **Post-hoc comparison after the Kruskal-Wallis test for the HRV changes within gender groups across time**

|  | Sample 1-Sample 2 | Test Statistic | Std. Error | Std. Test Statistic | Sig. | Adj. Sig. |
| --- | --- | --- | --- | --- | --- | --- |
| All participants | During the assessment-10 minutes before the assessment | 92.62 | 11.25 | 8.23 | < 0.01 | < 0.01 |
|  | During the assessment-10 minutes after the assessment | -106.71 | 11.56 | -9.23 | < 0.01 | < 0.01 |
|  | 10 minutes before the assessment-10 minutes after the assessment | -14.09 | 11.56 | -1.22 | 0.22 | 0.67 |
| Male | During the assessment-10 minutes before the assessment | 18.78 | 5.75 | 3.27 | < 0.01 | < 0.01 |
|  | During the assessment-10 minutes after the assessment | -23.82 | 5.88 | -4.05 | < 0.01 | < 0.01 |
|  | 10 minutes before the assessment-10 minutes after the assessment | -5.04 | 5.88 | -0.86 | 0.39 | 1.00 |
| Female | During the assessment-10 minutes before the assessment | 73.58 | 9.70 | 7.59 | < 0.01 | < 0.01 |
|  | During the assessment-10 minutes after the assessment | -82.76 | 9.98 | -8.29 | < 0.01 | < 0.01 |
|  | 10 minutes before the assessment-10 minutes after the assessment | -9.17 | 9.98 | -0.92 | 0.36 | 1.00 |

Each row tests the null hypothesis that the Sample 1 and Sample 2 distributions are the same.

Asymptotic significances (2-sided tests) are displayed. The significance level is 0.05.
