# Supplementary material for: Nursing students’ perceived anxiety and heart rate variability in mock skill competency assessment
Source: PLoS One. 2023 Oct 26;18(10):e0293509. doi: 10.1371/journal.pone.0293509 (PMC10602303; doi:10.1371/journal.pone.0293509)
Supplement: S2 Table — Each row tests the null hypothesis that the Sample 1 and Sample 2 distributions are the same. Asymptotic significances (2-sided tests) are displayed. The significance level is 0.05. (DOCX) [file pone.0293509.s005.docx]

**S2 Table.** **Post-hoc comparison after the Kruskal-Wallis test for the HRV in three performance-level groups across time**

|  | Sample 1-Sample 2 | Test Statistic | Std. Error | Std. Test Statistic | Sig. | Adj. Sig. |
| --- | --- | --- | --- | --- | --- | --- |
| T1, during the assessment | High performer-Medium performer | 2.33 | 7.05 | 0.33 | 0.74 | 1.00 |
|  | High performer-Low performer | 22.37 | 7.77 | 2.88 | < 0.01 | 0.01 |
|  | Medium performer-Low performer | 20.04 | 6.46 | 3.10 | < 0.01 | < 0.01 |
| T2, 10 minutes after the assessment | High performer-Medium performer | 7.88 | 6.47 | 1.22 | 0.22 | 0.67 |
|  | High performer-Low performer | 18.25 | 7.27 | 2.51 | 0.01 | 0.03 |
|  | Medium performer-Low performer | 10.37 | 6.27 | 1.65 | 0.10 | 0.30 |

Each row tests the null hypothesis that the Sample 1 and Sample 2 distributions are the same.

Asymptotic significances (2-sided tests) are displayed. The significance level is 0.05.
