# Supplementary material for: Nursing students’ perceived anxiety and heart rate variability in mock skill competency assessment
Source: PLoS One. 2023 Oct 26;18(10):e0293509. doi: 10.1371/journal.pone.0293509 (PMC10602303; doi:10.1371/journal.pone.0293509)
Supplement: S3 Table — Each row tests the null hypothesis that the Sample 1 and Sample 2 distributions are the same. Asymptotic significances (2-sided tests) are displayed. The significance level is 0.05. (DOCX) [file pone.0293509.s006.docx]

**S3 Table. Post-hoc comparison after the Kruskal-Wallis test for the HRV changes within three performance-level groups across three time points**

|  | Sample 1-Sample 2 | Test Statistic | Std. Error | Std. Test Statistic | Sig. | Adj. Sig. |
| --- | --- | --- | --- | --- | --- | --- |
| Low performer | During the assessment-10 minutes before the assessment | 19.23 | 5.97 | 3.22 | < 0.01 | < 0.01 |
|  | During the assessment-10 minutes after the assessment | -26.95 | 6.23 | -4.33 | < 0.01 | < 0.01 |
|  | 10 minutes before the assessment-10 minutes after the assessment | -7.72 | 6.23 | -1.24 | 0.22 | 0.65 |
| Medium performer | During the assessment-10 minutes before the assessment | 49.66 | 7.85 | 6.33 | < 0.01 | < 0.01 |
|  | During the assessment-10 minutes after the assessment | -58.47 | 8.10 | -7.22 | < 0.01 | < 0.01 |
|  | 10 minutes before the assessment-10 minutes after the assessment | -8.81 | 8.10 | -1.09 | 0.28 | 0.83 |
| High performer | During the assessment-10 minutes before the assessment | -24.35 | 5.52 | -4.41 | < 0.01 | < 0.01 |
|  | During the assessment-10 minutes after the assessment | 23.85 | 5.52 | 4.50 | < 0.01 | < 0.01 |
|  | 10 minutes before the assessment-10 minutes after the assessment | 0.50 | 5.52 | 0.09 | 0.93 | 1.00 |

Each row tests the null hypothesis that the Sample 1 and Sample 2 distributions are the same.

Asymptotic significances (2-sided tests) are displayed. The significance level is 0.05.
