# Supplementary material for: Nursing students’ perceived anxiety and heart rate variability in mock skill competency assessment
Source: PLoS One. 2023 Oct 26;18(10):e0293509. doi: 10.1371/journal.pone.0293509 (PMC10602303; doi:10.1371/journal.pone.0293509)
Supplement: Attachment — Submitted filename: Response to Reviewers.docx [file pone.0293509.s008.docx]

**Response to reviewers**

We thank the Editor and Reviewers for their time and consideration. We have modified the manuscript in response to the comments. For your convenience, the line and page numbers referenced below refer to the corresponding line and page numbers in the revised manuscript. The changes are highlighted in red in the manuscript, except for the part on changes resulting from editing.

**Response to Editor:**

Thank you very much for your comments. Our answers are as follows:

1. Please review your reference list to ensure that it is complete and correct. If you have cited papers that have been retracted, please include the rationale for doing so in the manuscript text, or remove these references and replace them with relevant current references. Any changes to the reference list should be mentioned in the rebuttal letter that accompanies your revised manuscript. If you need to cite a retracted article, indicate the article’s retracted status in the References list and also include a citation and full reference for the retraction notice.

Response to Editor comment No. 1: Thanks for your comments. We carefully checked the reference list and ensured that no retracted papers were cited. The reference list is complete.

**Response to Reviewer #2:**

The authors have considerably improved the manuscript, but there are still some points to be addressed:

Response: Thank you very much for your thorough review. Our responses are as follows:

1. Line 39: space missing (10min)

Response to Reviewer comment No. 1: We have now added the space between ‘10’ and ‘minutes’. Please refer to page 2, line 38.

2. Line 62: “Stress is a situation”. Revisit this definition – stress is not a situation. Definitions of stress and anxiety could be further elaborated upon/ contextualized.

Response to Reviewer comment No. 2: We have now revised the definition of stress. Further elaboration on students’ stress and anxiety was also added. Please refer to page 4, lines 61-70.

3. Line 94: “Additionally, evidence suggests that female students are more likely to focus their perceived stress on academic performance than male students [21].” This sentence is unclear. Are they more likely to attribute their stress to academic performance? Please clarify. In your response letter, you state gender differences to be a primary goal of your study – yet you do not discuss it much in depth in the introduction. What might be reasons for this gender difference? In what other domains can a gender difference be found. Also – are you referring to gender or sex differences? I presume the latter?

Response to Reviewer comment No. 3: We have now revised this sentence. Please refer to page 6, line 99.

As our secondary objective was to investigate the differences in perceived anxiety, HRV and performance between female and male students across (before, during and after) the mock skill competency assessment, we have now added more interpretations on sex differences. Please refer to page 6, lines 100-104.

Besides, we referred to sex differences in our study rather than gender differences. We have now revised the description. Please refer to page 7, line 121.

4. Line 97: “Alternatively” seems to be the incorrect connector. Alternatively to what?

Response to Reviewer comment No. 4: We have now revised the description. Please refer to page 6, line 105.

5. On page 6, you motivate the importance of this study in terms of incorporating stress measurement as a part of the assessment. What would be ethical implications of this? Would knowing that stress levels are being measured also possibly lead to increased anxiety?

Response to Reviewer comment No. 5: We agreed that it might lead to increased anxiety if students know that stress levels are being measured, especially when self-reported questionnaires are heavily relied on for measuring stress. As such, we attempted to analyse and interpret the changes in the cognitive and psychological states of students during the examination process by measuring the physio-psychological reactions. Physio-psychological response is an objective indicator, which can explain the state of the object under investigation at various levels without undue interference or influence on learning/examination. As an index to evaluate the physiological adaptability of the participants to a changing environment in skill assessment, the test of heart rate variability is recognised as useful and scientific in confirming the autonomic nervous system activity and balance level in objectively evaluating the psychological and emotional states of an individual [1]. In addition, students were required to arrive at the assessment venue 30 minutes before the assessment. They rested for at least 20 minutes before the measure, breathing spontaneously without talking.

Nonetheless, we have added the limitation and ethical implications due to the observational and monitoring effects of this study. Please refer to page 29, lines 527-528. “Fourth, it is possible that the Hawthorne effect [2] might have influenced the student’s anxiety and stress levels due to observational and monitoring effects”.

1. Yoo HH, Yune SJ, Im SJ, Kam BS, Lee SY. Heart Rate Variability-Measured Stress and Academic Achievement in Medical Students. Med Princ Pract. 2021;30(2):193-200. doi: 10.1159/000513781.
2. Sedgwick P, Greenwood N. Understanding the Hawthorne effect. BMJ. 2015;351:h4672. doi: 10.1136/bmj.h4672.
6. Line 137: “Monitoring physiological HRV-based stress in undergraduate nursing 138 students before the actual assessment may help build evidence on new ways to evaluate 139 students’ performance and overall professional qualities in nursing programmes.” In line with my previous comment, what are the ethical considerations of this statement?

Response to Reviewer comment No. 6: Thank you for noting this. We have now added the limitation and ethical implications due to the observational and monitoring effects of this study. Please refer to page 29, lines 527-528.

7. Line 305: I believe mean should be medians? The same goes for line 375. Wherever the statistics used were nonparametric, you should be reporting medians. Otherwise, Means and SDs.

Response to Reviewer comment No. 7: We have now changed ‘mean’ to ‘median’ in which nonparametric tests were used for data not normally distributed. Please refer to page 17, lines 311 and 313, page 18, line 335, and page 20 lines 368 and 371, page 21, line 373 and 376.

8. Formatting: (p > 0.05) (Table 2) >> (p > 0.05, Table 2)

Response to Reviewer comment No. 8: Thanks for noting this. We carefully checked the formatting issue and revised it accordingly. Please refer to page 17, lines 318-319, and page 20, line 362.

9. In the discussion, 2nd paragraph, you discuss the fact that there were no differences between anticipatory stress and stress during/ after the assessment. No differences were present between different levels of performers and anticipatory stress. Would the fact that this was a mock exam, with no real consequences, have led to these null results? I would be interested if there is any more literature out there on anticipatory stress, and possibly interactions with trait anxiety, for example.

Response to Reviewer comment No. 9: Thank you for highlighting this. We do discuss that no significant differences were reported 10 minutes before the assessment, however, significant differences were shown between the three groups during and 10 minutes after the assessment. Concerning the similar levels of anticipatory stress, we can find that the mock skill assessment stress exists and there are significant differences between the three groups during and 10 minutes after the assessment. We added further discussion about the potential reasons for the non-significant anticipatory stress between the three groups before the mock skill assessment. Please refer to page 23, lines 419-423.

10. When you say that previous research has yielded inconsistent findings, reiterate how they have been inconsistent (rather than referring to studies).

Response to Reviewer comment No. 10: Thank you for your comment. We have now added specific descriptions of the inconsistent findings from previous studies. Please refer to pages 24, lines 427-431.

11. Line 440 – “Testing HRV helps confirm the autonomic nervous system activity and sympathetic nervous system/parasympathetic nervous system balance.” This sentence is unclear. What do you mean by confirm?

Response to Reviewer comment No. 11: We have now further elaborated on this point. Please refer to pages 24-25, lines 443-453.

12. Line 445: “Therefore, students with stronger 445 autonomic nervous system responses (i.e., more significant reductions in HRV and 446 parasympathetic activity in response to assessments), and, suggestively, greater autonomic 447 flexibility, have a positive effect on higher performance [56].”

Response to Reviewer comment No. 12: Thanks for your comments. We further revised this sentence to make it clearer and coherent within the context. Hope the revised version with further elaborations can ease your concern. Please refer to pages 24-25, lines 446-453.

13. Line 447: future studies

Response to Reviewer comment No. 13: We have now revised the description. Please refer to page 25, line 453.

14. Line 551 – “First, the current findings 552 could only indicate the one-way possible causal relationship between HRV and performance; 553 that is, higher stress (lower HRV) could contribute to higher performance.” If you only tested for differences in groups, how can the relationship be causal?

Response to Reviewer comment No. 14: We have now deleted ‘causal’ for the relationship between HRV and performance. Please refer to page 30, line 557.

15. Line 559 – “Further studies may dig data into analysing” – dig data?

Response to Reviewer comment No. 15: We have now revised the description. Please refer to page 31, line 564.

16. A clarification/ discussion of what “optimal” stress levels is necessary.

Response to Reviewer comment No. 16: Thanks for your comments. A clarification of an optimal stress level was added. Please refer to page 31, lines 567-570. We also highlighted the significance of an optimal level of stress in the Background. Please refer to page 7, lines 123-127.

17. In terms of the scientific and societal motivation of this study, I find the view presented is at times too narrow. I think it is important how these findings might generalize elsewhere. Instead of focusing so much on how this is relevant for nursing assessment, I think it is also important to motivate the importance of your study from a more fundamental perspective.

Response to Reviewer comment No. 17: We do agree that highlighting the importance of this study is needed, not only from the perspective of highlighting the importance of HRV measurement applied in nursing curriculums. Hence, we not only highlighted the importance of developing wellness initiatives (e.g. stress management and resilience promotion) in health professional education programme (page 29, lines 536-541), but we also added implications to help understand the mechanism of stress response in student population. Please refer to page 30, lines 549-555.

18. With regards to my point concerning findings pertaining to gender in the discussion – I still suggest you remove any speculation, especially considering your findings were insignificant.

Response to Reviewer comment No. 18: Thank you for highlighting this. We have now deleted the discussion on non-significant sex differences in perceived anxiety and HRV before and after the assessment.
